# Editorial of Special Issue “Deep Learning and Machine Learning in Bioinformatics”

**DOI:** 10.3390/ijms23126610

**Published:** 2022-06-14

**Authors:** Mingon Kang, Jung Hun Oh

**Affiliations:** 1 Department of Computer Science, University of Nevada, Las Vegas, NV 89154, USA; mingon.kang@unlv.edu; 2 Department of Medical Physics, Memorial Sloan Kettering Cancer Center, New York, NY 10065, USA

In recent years, deep learning has emerged as a highly active research field, achieving great success in various machine learning areas, including image processing, speech recognition, and natural language processing, and now rapidly becoming a dominant tool in biomedicine [1]. In particular, a dramatically increasing number of deep learning-based approaches have been proposed in biomedical image analysis and biosignal processing, as well as medical prediction modeling. However, the application of deep learning to genomics and bioinformatics has been rather limited, perhaps due to the combined difficulties of interpretation as well as steep data requirements.

One of the major challenges is that many approaches in deep learning and traditional machine learning are based on the assumption that the number of samples is huge in order to train models with a vast number of features. The situation in medicine is often reversed by necessity: the number of features desired to be analyzed is often one or two orders of magnitude greater than the number of samples. Researchers must contend with this fundamental issue, and in the end must be content with models that are consistent with the data.

In this Special Issue entitled “Deep Learning and Machine Learning in Bioinformatics”, submissions address the application of deep learning and novel machine learning methods to diverse bioinformatic problems and provide practical guidance. These methods include useful approaches that may improve predictive performance and separately enhance our understanding of biological mechanisms of target diseases.

Among the 55 submissions reviewed, 21 were accepted, including 17 research articles and 4 reviews, with 124 contributors. The contributions were global, for the accepted papers originating from 12 countries, including Australia (2), China, France, Italy (3), Japan (2), Poland, South Korea (2), Spain, Sweden, Taiwan, Thailand, and the United States (5). Figure 1 shows the map of countries with the symbol ★ for the first or corresponding authors of the accepted papers.

Ten research papers demonstrated the application of deep learning to various kinds of biological data. Le et al. proposed an ensemble neural network to identify essential genes via word embedding features from genomic sequences [2]. Persson Hodén et al. developed a convolutional neural network (CNN) model capable of efficiently identifying true mRNA cleavage sites, which was implemented as an R package called smartPARE [3]. Nosi et al. proposed a neural network method to detect MET exon 14 skipping events using RNAseq data from The Cancer Genome Atlas (TCGA) archive for lung cancer [4]. Alessandri et al. developed a new autoencoder model, called Sparsely Connected Autoencoders, to improve the traditional decoder model for better identifying biological features from single cell data [5]. Al Mamun et al. developed a multi-run concrete autoencoder to identify a stable set of features which was applied to TCGA genome-wide lncRNA expression profiles in 12 cancers, resulting in the identification of key lncRNAs [6]. Lee et al. introduced a peptide data augmentation method, which was employed to predict spider neurotoxic peptides, showing improved predictive power when coupled with a CNN model [7]. Madani et al. developed a novel deep learning sequence-based solubility predictor, called DSResSol, for fast, reliable, and inexpensive prediction of protein solubility [8]. Zulfiqar et al. developed a 1D CNN-based model, named Deep-4mCGP, to identify 4mC sites in Geobacter pickeringii [9]. Roethel et al. developed a deep learning architecture for a holistic sequential and structural analysis of biomolecules [10].

Hazra et al. employed generative adversarial networks (GAN) to create synthetic nucleic acid sequences of the cat genome [11].

Seven research papers used traditional (non-deep learning) machine learning approaches to analyze biological data. Two computational methods were introduced, PUP-Fuse [12] and PredNTS [13], for the prediction of pupylation sites and nitrotyrosine sites, respectively, by integrating multiple sequence representations coupled with a random forest approach. Rodin et al. proposed a novel computational pipeline to dissect the response to cancer immunotherapy, employing systems biology and Bayesian network techniques on flow cytometry data [14]. Campos et al. employed machine learning approaches to identify essential genes common to both Caenorhabditis elegans and Drosophila melanogaster [15]. Charoenkwan et al. developed a sequence-based predictor, named iBitter-Fuse, to identify bitter peptides by fusing multi-view features [16]. Jabeen et al. adopted a random forest model to identify novel high activity agonists of human ectopic olfactory receptors [17]. Pouryahya et al. proposed a network-based clustering method coupled with optimal mass transport theory to predict cell line-drug sensitivity, and showed that random forest modeling conducted on the resulting cell line-drug clusters outperformed alternative computational methods in predicting in vitro drug responses [18].

Four papers reviewed the use of deep learning or machine learning approaches to biological data analysis. Auslander et al. reviewed machine learning/deep learning approaches incorporated to establish bioinformatics and computational biology frameworks in the areas of molecular evolution, protein structure analysis, systems biology, and disease genomics [19]. Del Giudice et al. comprehensively reviewed machine learning/deep learning solutions for computational problems in bulk and single-cell RNA-sequencing data analysis [20]. Banegas-Luna et al. discussed the interpretability of machine learning/deep learning methods in cancer research [21]. Defresne et al. reviewed deep learning methods used for protein design [22].

In summary, the articles in this Special Issue provide a range of reviews and updates to the use of deep learning and machine learning in bioinformatics.

## Figures and Tables

**Figure 1:**
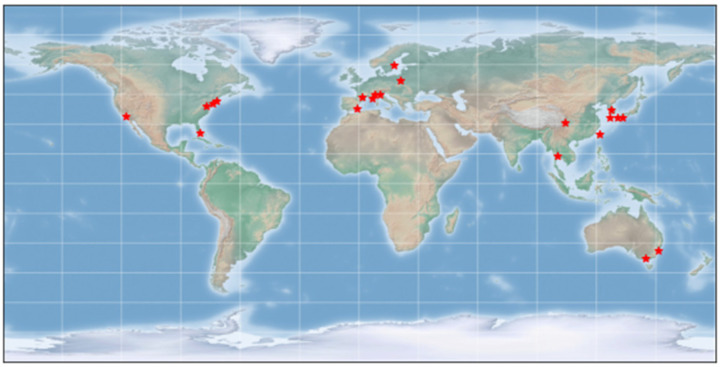
A map of countries with the symbol ★ for the first or corresponding authors of the accepted papers.
